# Evaluation of root and canal morphology of mandibular premolar amongst Saudi subpopulation using the new system of classification: a CBCT study

**DOI:** 10.1186/s12903-023-03002-1

**Published:** 2023-05-15

**Authors:** Mohmed Isaqali Karobari, Azhar Iqbal, Jamaluddin Syed, Rumesa Batul, Abdul Habeeb Adil, Sara Akili Khawaji, Mohammed Howait, Osama Khattak, Tahir Yusuf Noorani

**Affiliations:** 1 Department of Restorative Dentistry & Endodontics, Faculty of Dentistry, University of Puthisastra, Phnom Penh, 12211 Cambodia; 2 Department of Restorative Dentistry, College of Dentistry, Jouf University, Sakaka, 72345 Saudi Arabia; 3 Oral Basic and Clinical Sciences, Faculty of Dentistry, King Abdulaziz University, p.o box 80209, Jeddah, Saudi Arabia; 4 Conservative Dentistry Unit, School of Dental Sciences, Universiti Sains Malaysia, Health Campus, Kubang Kerian, Kota Bharu, Kelantan 16150 Malaysia; 5 Department of Community Dentistry, School of Dental Sciences, Universiti Sains Malaysia, Health Campus, Kubang Kerian, Kota Bharu, Kelantan 16150 Malaysia; 6 Department of Endodontics, Faculty of Dentistry, King Abdulaziz University, p.o box 80209, Jeddah, Saudi Arabia; 7 Department of Conservative Dentistry & Endodontics, Saveetha Institute of Medical and Technical Sciences University, Chennai 600077, Tamil Nadu, India

**Keywords:** CBCT, Dental anatomy, Dental diagnostic imaging, Dental pulp, Endodontics, Morphology, Root, Root canal, Premolars

## Abstract

**Background:**

The clinician should have complete knowledge of the normal anatomy of the root as well as complexities in the root canal configuration for a better outcome, as missed or improper handling of the canal system can lead to the failure of an entire endodontic procedure. The present study aims to assess the morphology of roots and canals in permanent mandibular premolars in the Saudi subpopulation with a new classification system.

**Methods:**

The present study includes 1230 mandibular premolars (645 first premolars and 585-second premolars) from 500 CBCT images of the patients, including retrospective data. iCAT scanner system (Imaging Sciences International, Hatfield, PA, USA) was used to obtain the images; scanning of 8 × 8 cm images was performed at 120 KVp and 5–7 mA with a voxel size of 0.2 mm. The new method of classification presented by Ahmed et al. 2017 was used to record and classify the root canal morphology, followed by recording the differences regarding the age and gender of the patients. Comparison of canal morphology in lower permanent premolars and its association with gender and age of the patients was done by Chi-square test/ Fisher exact test; the significance level was set at 5% (p ≤ 0.05).

**Results:**

The left mandibular 1st and 2nd premolars with one root were 47.31%, with two roots were 2.19%. However, three roots (0.24%) and C-shaped canals (0.24%) were reported only in the left mandibular 2nd premolar. The right mandibular 1st and 2nd premolars with one root were 47.56%, with two roots were 2.03%. The overall percentage of the number of roots and canals in the first and second premolars ^1^ PM ^1^ (88.38%), ^2^ PM ^1^ B ^1^ L ^1^ (3.5%), ^2^ PM B ^1^ L ^1^ (0.65%), ^1^ PM ^1–2−1^ (3.08%), ^1^ PM ^1–2^ (3.17%), ^1^ PM ^1–2−1–2^ (0.24%), ^3^ PMMB ^1^ DB ^1^ L^1^ (0.48%). However, the C-shaped canals (0.40%) were reported in right and left mandibular second premolars. No statistically significant difference was reported between mandibular premolars and gender. A statistically significant difference was reported between mandibular premolars and the age of the study subjects.

**Conclusion:**

Type I (^1^ TN ^1^) was the major root canal configuration in permanent mandibular premolars, which was higher among males. The CBCT imaging provides thorough details about the root canal morphology of lower premolars. These findings could support diagnosis, decision-making, and root canal treatment, for dental professionals.

## Background

The success of endodontic treatment mainly relies on locating the canals, complete debridement, obturation and ultimately sealing the canals in three dimensions with an appropriate sealer [1]. The practitioner should have complete knowledge of the normal anatomy of the root as well as complexities in the root canal configuration for a better outcome, as missed or improper handling of the canal system can lead to the failure of an entire endodontic procedure [2]. Although permanent lower premolars generally have a single root and one canal system, these are considered the most challenging teeth due to their diverse variations in the root canal system and the high frequency of multiple canals or roots [3]. However, the first premolar can have two roots (1.8%), three roots (0.2%), and rarely 4 roots (< 0.1%). It consists of a single canal (75.8%) or more than two canal systems (24.2%), and apical foramen can be one or multiple [4].

Similarly, the second premolar is extremely complex in its root anatomy and the factors responsible for these variations are ethnicity, gender, age, genetics environmental and geographical differences [5]. The majority of the second premolar has a single root (99.6%), canal, and apical foramen. The description of the mandibular first premolar tooth in the textbooks is typical of a tooth with a single root. It has also been stated that there are variations with two roots, three roots, and even four roots; however, these are quite uncommon [6]. It can be concluded that the mandibular first premolar has a higher incidence of altered morphology of the root and canal compared to the second premolar [7]. Hence absolute insight into the root canal system is essential and root canal morphology classification plays a crucial role in documenting and assisting the practitioner to achieve the desired outcome of an endodontic treatment [8].

The root and canal morphology is analyzed by numerous methods like canal staining, sectioning, clearing technique, and conventional radiographic technique. Further, micro-computed tomography (Micro CT) a 3D imaging method, digital radiography, and spiral computed tomography can also be used [9, 10]. CBCT (cone beam computed tomography) is employed in this study to evaluate the morphology of the root and canal in permanent mandibular premolars. It is a widely used technique in dentistry since its introduction in 1990 and has been applied in various dental fields considering its advantageous and non-invasive nature, which provides minute details of the internal and external anatomy of the tooth and its surroundings without interfering with the structure of the tooth and its morphology [1, 11]. It provides a three-dimensional view with lesser radiation exposure and higher resolution allowing to study of the details of the anatomical structure in both quantitative and qualitative aspects thoroughly [12, 13].

Many authors have given several root canal classifications in dentistry where Weine et al. was considered the first one to classify the canal system. Further Vertucci et al. reported the complex configuration of root canals into 8 types which state the pattern of classification from the pulp chamber in the primary canal to the apex of the root [8, 14]. However, this classification holds a few drawbacks such as mandibular premolars with two roots are not classified separately whereas the mesial and distal roots of lower molars were considered separately [15]. A new classification system was presented by Ahmed et al. in 2017 which includes coding of tooth numbers, and roots followed by coding the configuration of root canals. It allowed the accurate categorization of complex root canal systems and it also conquered the odds of previous classifications which could not recognize many root canals in an appropriate categories [15, 16].

Numerous studies are conducted among the Saudi population indicating that the majority of first and second permanent mandibular premolars have one root, one canal, and one apical foramen. However, two or more roots with more than one canal configuration and apical foramen were also noticed [17–19]. Hence complete understanding of root canal morphology with an appropriate classification is the key to the success of an endodontic procedure and thus, to avoid any unfavorable outcomes [20]. To the best of the author’s knowledge, no study is conducted where root canal morphology of permanent lower premolars is performed using a new classification system in the Saudi subpopulation using the CBCT technique. The present study aims to assess the root canal morphology of permanent mandibular premolars in the Saudi subpopulation with a new classification system.

## Methods

### Ethical consideration

Ethical approval was obtained from the Local Committee of Bioethics for Research at the dentistry college, King Abdul-Aziz University, with an Ethical Approval No. 025-02-22. Informed consent, due to the retrospective nature of the study, was yielded by the Committee of Bioethics for Research, College of Dentistry, King Abdul-Aziz University, Jeddah, Saudi Arabia. However, a sign from the patient on a general consent form was taken before any investigation or treatment. This included signing a consent form so that findings can be used in the future without revealing any personal information of the participants for retrospective studies.

### Sample size

One-hundred eighty-three (183) scanned samples were regulated by the G power 3.1.9.4 software with x2 test, the goodness-of-ft test was a statistical test, and power analysis was A priori. The needed sample size was calculated given α, power, and effect size. The present study comprises 1230 mandibular premolars (645 first premolars and 585-second premolars) from 500 CBCT images of the patients, including from retrospective data.

### Data collection

To access patient records that included information on age and gender, prior approval from the vice dean of academic and clinical affairs was required. The radiology department of the College of Dentistry provided access to the CBCT images, which were acquired for use unrelated to this investigation. To preserve any personally identifiable information of CBCT scans that would conflict with the ethical obligations of the patient’s data, the name of the patient and other personal information was not recorded; the study team was restricted to the recorded data. The study included CBCT images of the lower premolars. Inclusion criteria were the healthy images of lower premolars with small carious or restorative crowns, fully formed root apex and defects-free radiographic images. Whereas exclusion criteria were root canal-treated teeth, fractured upper and lower posterior teeth, post and core, calcification, resorption defects, and anomalies of crown and root.

### Calibration

The calibration of the present study was carried out by an expert endodontist and an observer. The spectator was guided to study the 50 CBCT images. The root canal morphology was identified by evaluating the images of CBCT by utilizing axial, sagittal, and coronal views, and a single score was assigned to each tooth. After adequate deliberation and discussion on disagreements, a harmonious decision was taken.

### Root and canal analysis

**Fig. 1:**
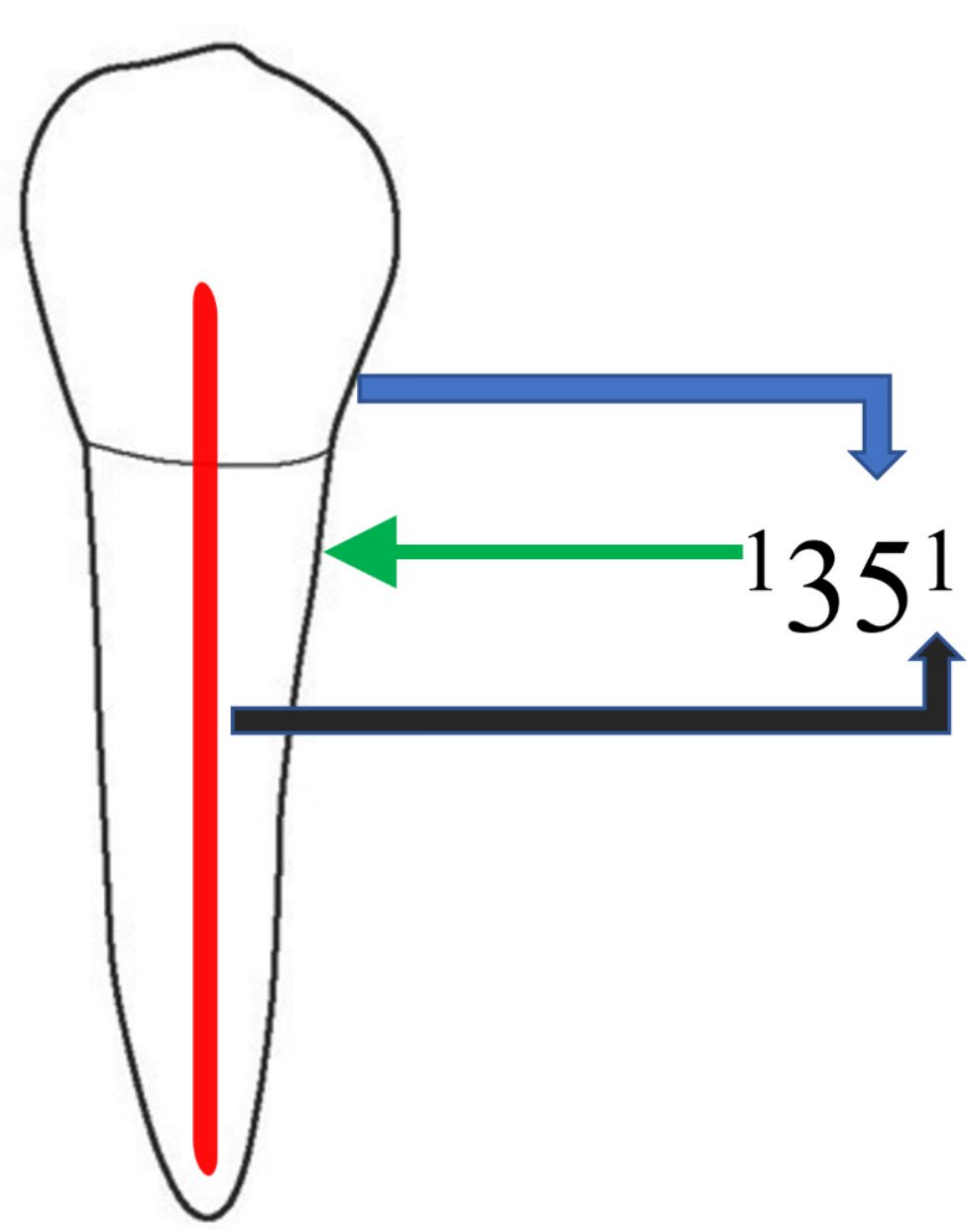
New system of classification for the morphology of root canal of left mandibular first premolar classified with the new system of classification, narrated as code ^1^35^1^. The code is consisting of three components, first is the tooth number represented by the blue arrow, the second is number of roots symbolized by the green arrow and the third is the configuration of the canal depicted by the black arrow. A superscript before the tooth number is written which indicates the number of roots, so it is one root with tooth number (35). Details about the configuration of the canal are displayed as superscripts after the tooth number, during the passage of the root canal starting from the orifices [O], passing through the canal [C], and ending by the foramen [F], so it is one canal

The images were obtained and analyzed by an expert endodontist and an observer using the iCAT scanner system (Imaging Sciences International, Hatfield, PA, USA); scanning of 8 × 8 cm images was performed at 120 KVp and 5–7 mA with a voxel size of 0.2 mm. Images have been viewed and set on a calibrated Dell (Round Rock, TX) 17-inch (0.28 dot pitch) high-resolution monitor at 16-bit color depth and 1024 × 768 pixels of screen resolution. Different age groups (10 to 20, 21 to 30, 31 to 40, 41 to 50, 51 to 60, and 61 years above) were assigned to divide the obtained images. The patients were divided into males or females depending on gender. The root canal morphology was recorded and classified with the new system of classification presented by Ahmed et al. 2017 (Fig. 1) and the differences concerning the age and gender of the subjects were noted.

### Statistical analysis

SPSS 26th version of the software (Armonk, NY, USA, IBM SPSS Statistics) performed the statistical analysis. Descriptive statistics, like mean frequency and standard deviation, were carried out. Comparison of root canal morphology in lower premolars and its association with patient’s age and gender was accomplished by the Chi-square test/ Fisher exact test; the significance level was set at 5% (p ≤ 0.05).

## Results

**Fig. 2:**
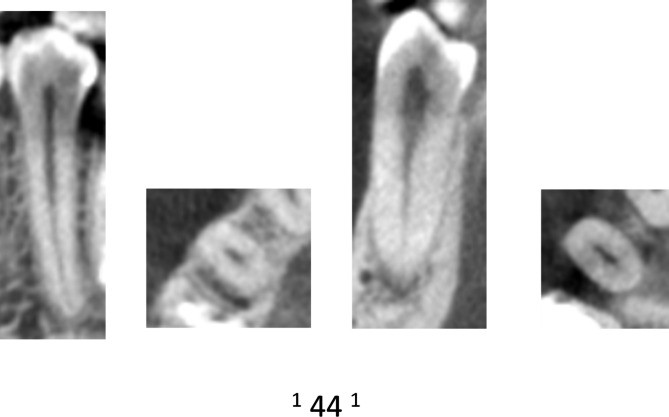
CBCT View (Sagittal and axial) of right mandibular first premolar showing the code ^1^PM^1^

**Fig. 3:**
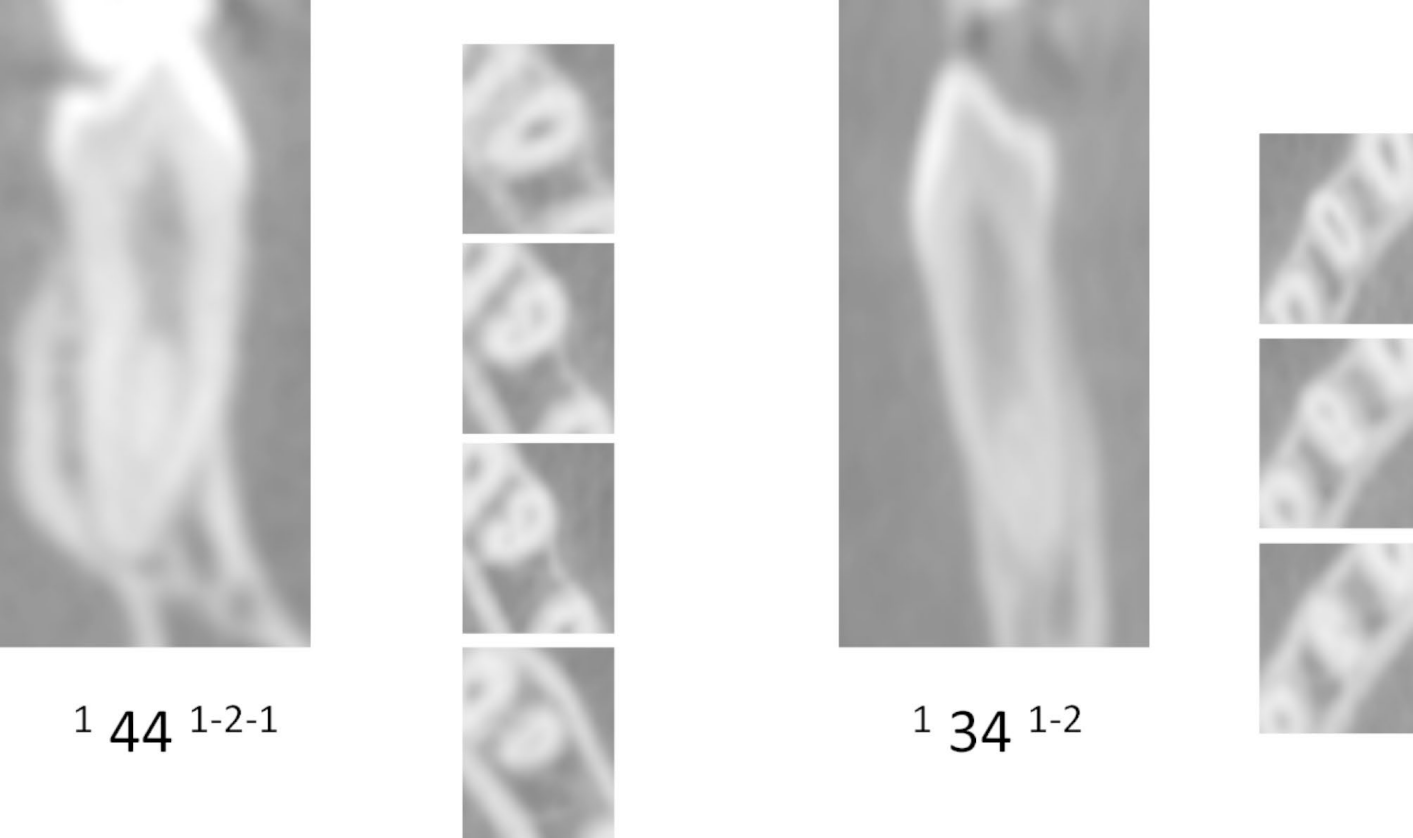
CBCT View (Sagittal and axial) of mandibular first premolars showing the canal variations

**Fig. 4:**
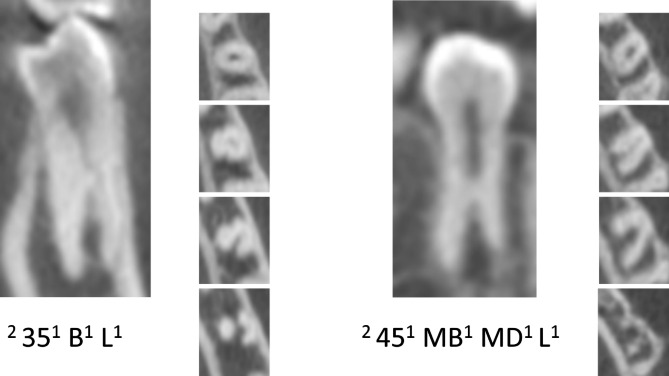
CBCT View (Sagittal and axial) of mandibular second premolars showing the root and canal variations

Table 1 displays the distribution of mandibular premolars according to Ahmed’s classification. The total number of first and second mandibular premolars that were included in the current study was 1230. The majority of left mandibular premolars were with ^1^ TN ^1^ (88.14%) configuration followed by ^2^ TN ^1^ B ^1^ L ^1^ (3.42%). While the majority of right mandibular premolars were with ^1^ TN ^1^ (89.31%) configuration followed by ^2^ TN ^1^ B ^1^ L ^1^ (3.42%) Figs. 2, 3 and 4. The overall percentage of the number of roots and canals in the first and second premolars ^1^ PM ^1^ (88.38%), ^2^ PM ^1^ B ^1^ L ^1^ (3.5%), ^2^ PM B ^1^ L ^1^ (0.65%), ^1^ PM ^1–2−1^ (3.08%), ^1^ PM ^1–2^ (3.17%), ^1^ PM ^1–2−1–2^ (0.24%), ^3^ PMMB ^1^ DB ^1^ L^1^ (0.48%). However, the C-shaped canals (0.40%) were reported in right and left mandibular second premolars.

**Table 1:** Distribution of root and root canal morphology of mandibular premolars

Classification	Left 1st PM		Right 1st PM		Left 2nd PM		Right 2nd PM		Overall
	n	%	n	%	n	%	n	%	n (%)
^1^ PM ^1^	262	40.62	264	40.93	278	47.52	283	48.38	1087 (88.37)
^2^ PM ^1^ B ^1^ L ^1^	21	3.25	21	3.25	1	0.17	1	0.17	44 (3.5)
^2^ PM B ^1^ L ^1^	3	0.46	3	0.46	2	0.34			8 (0.65)
^1^ PM ^1–2−1^	18	2.79	16	2.49	2	0.34	2	0.34	38 (3.08)
^1^ PM ^1–2^	18	2.79	16	2.49	3	0.51	2	0.34	39 (3.17)
^1^ PM ^1–2−1–2^	1	0.15	2	0.32					3 (0.24)
^3^ PMMB ^1^ DB ^1^ L^1^					3	0.51	3	0.51	6 (0.48)
C-Shaped canals					3	0.51	2	0.34	5 (0.40)
Total	323	50.06	322	49.94	292	49.9	293	50.1	1230 (100)

PM- premolars

Table 2 illustrates the distribution of mandibular premolars according to their root numbers. The left lower 1st and 2nd premolars with single roots were 47.31%, with two roots were 2.19%. However, three roots (0.24%) and C-shaped canals (0.24%) were reported only in the left mandibular 2nd premolar. The right mandibular 1st and 2nd premolars with one root were 47.56%, with two roots were 2.03%. However, three roots (0.24%) and C-shaped canals (0.16%) were reported only in the right mandibular 2nd premolar. The overall percentage of one root was 94.87%, two roots (4.22%), three roots (0.48%), and C-shaped canals (0.40%) respectively.

**Table 2:** Distribution of mandibular premolars according to number of roots

Number of roots	Left 1st PM	Left 2nd PM	Right 1st PM	Right 2nd PM	Overall n (%)
One root	299	283	298	287	1167 (94.87)
Two roots	24	3	24	1	52 (4.22)
Three roots	0	3	0	3	6 (0.48)
C-shaped	0	3	0	2	5 (0.40)
Total	323	292	322	293	1230 (100)

PM- premolars

Table 3 demonstrates the distribution of the left mandibular 1st and 2nd premolar concerning gender. The distribution of left mandibular 1st and 2nd premolar was higher among males compared to females. No statistically significant difference was reported between the left mandibular 1st and 2nd premolar and gender of the study subjects with p-value > 0.05.

**Table 3:** Distribution of left mandibular 1st and 2nd premolar with respect to gender

Classification	Left 1st PM			Left 2nd PM		
	Male	Female	p-value	Male	Female	p-value
^1^ PM ^1^	139	122	0.056	157	120	0.528
^1^ PM ^1–2−1^	14	4		2	0	
^1^ PM ^1–2^	12	6		1	2	
^1^ PM ^1–2−1–2^	0	1				
^2^ PM ^1^ B ^1^ L ^1^	17	4		0	1	
^2^ PM B ^1^ L ^1^	2	1				
^3^ PMMB ^1^ DB ^1^ L ^1^				2	1	
C-Shaped canals				3	0	
Total	184	138		165	124	

PM- premolar, *significant value < 0.05; Chi-square test

Table 4 illustrates the distribution of the right mandibular 1st and 2nd premolar concerning gender. The distribution of the right mandibular 1st and 2nd premolar was higher among males compared to females. No statistically significant difference was reported between the right mandibular I^st^ and 2nd premolar and gender of the study subjects with p-value > 0.05.

**Table 4:** Distribution of right mandibular 1st and 2nd premolar with respect to gender

Classification	Right 1st PM			Right 2nd PM		
	Male	Female	p-value	Male	Female	p-value
^1^ PM ^1^	138	135	0.293	160	122	0.596
^1^ PM ^1–2−1^	12	4		2	0	
^1^ PM ^1–2^	9	7		1	2	
^1^ PM ^1–2−1–2^	1	1				
^2^ PM ^1^ B ^1^ L ^1^	16	5		0	1	
^2^ PM B ^1^ L ^1^	2	1				
^3^ PMMB ^1^ DB ^1^ L ^1^				2	1	
C-Shaped canals				2		
Total	178	153		167	123	

PM- premolar, *significant value < 0.05; Chi-square test

Table 5 demonstrates the left mandibular 1st and 2nd premolar concerning the age of the study subjects. The distribution of left mandibular 1st premolar with ^1^ TN ^1^ classification was higher among 31–40 years than 21–30 years of study subjects. whereas the distribution of left mandibular 2nd premolar with ^1^ TN ^1^ classification was higher among 21 to 30 years, followed by 31 to 40 years of age of the study subjects. However, a statistically significant difference was reported between the left mandibular I^st^ and 2nd premolar and age of the study subjects with p-value < 0.05.

**Table 5:** Distribution of left mandibular 1st and 2nd premolars with respect to age

Classification	Left 1st PM							Left 2nd PM						
	AGE						p-value	AGE						p-value
	10–20	21–30	31–40	41–50	51–60	60+		10–20	21–30	31–40	41–50	51–60	60+	
^1^ PM ^1^	18	65	79	43	31	26	0.001*	23	80	77	43	30	25	0.001*
^1^ PM ^1–2−1^	1	8	7	2	0	0		0	0	1	1	0	0	
^1^ PM ^1–2^	4	4	7	1	0	2		1	1	0	0	0	1	
^1^ PM ^1–2−1–2^	0	0	1	0	0	0								
^2^ PM ^1^ B ^1^ L ^1^	2	7	5	4	1	2		0	0	1	0	0	0	
^2^ PM B ^1^ L ^1^	1	2	0	0	0	0		0	1	0	1	0	0	
^3^ PMMB ^1^ DB ^1^ L 1								0	2	1	0	0	0	
C-Shaped canals								0	1	2	0	0	0	
Total	26	86	99	50	32	30		24	85	82	45	30	26	

PM- premolar, *significant value < 0.05; Chi-square test

Table 6 illustrates the distribution of the right mandibular 1st and 2nd premolar concerning the age of the study subjects. The distribution of right mandibular 1st and 2nd premolar with ^1^ TN ^1^ classification was higher among 31–40 years, followed by 21–30 years of age of the study subjects. However, a statistically significant difference was reported between the left mandibular 1st and 2nd premolar and the age of the study subjects with a p-value < 0.05.

**Table 6:** Distribution of right mandibular 1st and 2nd premolars with respect to age

Classification	Right 1st PM							Right 2nd PM						
	AGE						p-value	AGE						p-value
	10–20	21–30	31–40	41–50	51–60	60+		10–20	21–30	31–40	41–50	51–60	60+	
^1^ PM ^1^	16	67	80	42	33	26	0.001*	24	84	85	39	27	24	0.001*
^1^ PM ^1–2−1^	2	5	6	2	0	1		0	0	1	1	0	0	
^1^ PM ^1–2^	4	5	4	1	0	2		1	1	0	0	0	1	
^1^ PM ^1–2−1–2^	0	0	1	0	0	1								
^2^ PM ^1^ B ^1^ L ^1^	1	8	5	5	1	1		0	0	1	0	0	0	
^2^ PM B ^1^ L ^1^	1	2	0	0	0	0								
^3^ PMMB ^1^ DB ^1^ L ^1^								0	2	1	0	0	0	
C-Shaped canals									1	1				
Total	24	87	96	50	34	31		25	88	89	40	27	25	

PM- premolar, *significant value < 0.05; Chi-square test

## Discussion

In clinical endodontics, diagnostic imaging is important. The CBCT images could be valuable for root and canal diagnosis, as well as future endodontic treatment approaches [21]. Root canal treatment is considered to be successful when all the root canals of a tooth are explored, cleaned, debrided, shaped, and completely obturated [22]. The causes of root canal failure are untreated canals, impaired disinfection, and incomplete obturation. As a result, appropriate clinical and radiographic assessment is required for root canal therapy to be successful [17]. The CBCT is the radiographic technique that is used for the diagnosis and preoperative as well as postoperative assessment of root canals of involved teeth [23]. The major advantage of CBCT over other imaging techniques is that it provides a 3D image of the interested object with lesser radiation exposure of a smaller area and superior quality of image [24]. CBCT is the well dependable tool to interpret the anatomy and morphology of root canals, and assessment of postoperative complications of the procedure like overfilled canals, broken instruments, root perforation, fractures, and calcification of root canals. As avoidance and failure to treat all canals can negatively impact the outcome of treatment and hence CBCT greatly helps in deceiving the unfortunate mistakes and improves the overall prognosis of an endodontic treatment [24, 25].

Regarding the root and canal morphology of teeth, there is a great variation in the literature, and the human lower premolars are no exception [6]. Human teeth anatomy is complex when it comes to the root numbers, canals, and their shape which varies greatly. The factors responsible for these variations are age, gender, ethnicity, sex, and study design [4]. Hence a classification system is required to classify the root canals accordingly and avoid procedural errors. Weine et al. was the first person to classify root canals in 1969. He categorized the single root from the pulp chamber to the apex of the root into four types. Later Vertucci et al. 1974 introduced another system of classification and along with him, many other classifications were developed [26–29]. But these classification systems fail to classify many canals configuration as the anatomy of human teeth is extremely variable and hence many root canals of the teeth remained unclassified. For example, an identical number of canals with different numbers of roots of maxillary premolars were categorized under the same type as per Vertucci’s classification. Similarly, mandibular premolars with the same canal configuration but varying root numbers were also put under the same type. In contrast to this, the canals of the mesial and distal roots of the mandibular molar were classified separately by Vertucci’s classification [15, 26, 30].

A new system of classification was introduced by Ahmed et al. which precisely and accurately described the canal configuration and provided complete information about the root numbers, canals, and accessory canals. It successfully classified the complex anatomy of teeth regardless of any anomaly or presence of single or multi-rooted teeth which could not be addressed by previous classification methods [15, 26, 31–33]. It has basic principles for classifying the canals which include coding the tooth number by using any tooth numbering system and then writing the number of roots and canal configurations. Hence it is successfully used clinically among the practitioner as well as among students to classify the root canal system [1, 9, 16, 34]. Considering the above-mentioned advantages of CBCT and the new classification system, this study aims to classify the mandibular first and second premolar using a new classification system in the Saudi subpopulation applying the CBCT technique.

The single root and root canal is the usual morphology of lower premolars but they are considered to be the most difficult teeth to treat as the canal configuration of mandibular premolars differs significantly concerning age, gender, ethnicity, and race [4, 35]. The total number of 1st and 2nd mandibular premolars included in the study is 1230 which were classified according to the new classification system. As per the author’s knowledge, there is no other study conducted among the Saudi subpopulation where a new system of classification is used to classify the mandibular premolars.

The current study includes CBCT scans from 1230 patients, yielding a large number of teeth for comparison regarding tooth classification and sex distributions of the various morphologies. The results reported in a Saudi population for root and root canal morphology were substantially comparable to prior research in various populations [4, 36–40]. The most common canal configuration of mandibular first and second premolars reported in our study was ^1^ TN ^1^ then ^2^ TN ^1^ B ^1^ L ^1^. This is in accordance with other studies conducted on the Saudi population using Vertucci classification [17, 20] and similar canal configuration was found in different population like South African, Jordanian, Turkish, and Iranian [5, 38, 41, 42]. Although a higher number of lower first and second premolars were ^1^ TN ^1^ and ^2^ TN ^1^ B ^1^ L ^1^, we discovered that the mandibular first premolars were also ^1^ TN ^1–2−1^ and ^1^ TN ^1–2^ respectively. These results highlight how crucial it is to locate the second root canal in the lower first premolars to complete the cleaning and obturation of the root canal system. The majority of mandibular first and second premolars both on the left and right sides had one root followed by two roots. Clearly right and left first mandibular premolars had more incidence of two roots compared to the second premolar and then followed by three rare roots. Hence it can be concluded that the mandibular first premolar varies more in anatomy when compared to the mandibular second premolar. This result is in accordance with different studies of the Saudi population [2, 17, 43]. However, it is noted that the occurrence of two roots in the mandibular first premolar is high in the Saudi population when compared with other individuals like Turkey, Iran, Jordan, and Egypt [41, 44–46]. Another study on the Kuwaiti population [47] reported an increased presence of two roots in the first (24.9%) and the second mandibular premolars (20.8%). All these variations are mostly due to different ethnicity, race, and study design.

The current study also showed the presence of C-shaped canals only in the lower second premolar of the right and left sides. They are minor in number and this result is in accordance with the previous studies [38, 48, 49]. In contrast to this Venezuelan population [50] presented a higher incidence of the canal which are C shaped in the lower first premolar (28.94%) and second premolar (7.14%). Astonishing to the above findings the studies of Saudi [17] and Iranian populations [42] had no C-shaped canals in their samples. It is an unusual anatomy of premolars which is supposed to happen due to lack of formation of dentine on the lingual side or it can also occur if Hartwig’s epithelial root sheath fails to fuse during the stage of tooth development. Hence it is challenging work to clean and achieve successful results of the treatment as these canals are present with the thinness dentinal walls, narrow canals, and concavities on the roots [49, 50]. The likelihood of a C-shaped canal in lower second premolars was suggested by this prevalence. It is advised to utilize magnification and instrumentation with an anti-curvature method when treating mandibular premolars with a C-shaped root canal since these teeth have small canals, concave roots, and thin dentinal walls in the lingual zone close to the mesial area. Therefore, the mandibular second premolars with several canals should be endodontically treated as a C-shaped root canal to prevent failure of the treatment [51]. Henceforth it is evident that mandibular premolars differ remarkably in their anatomy and notably affect the outcome of endodontic procedure.

Gender difference is another factor that determines the differences in root canal morphology. In the current study, males have a higher distribution of first and second premolars (both sides) than females. This conclusion is in harmony with the previous studies of the Saudi population [2, 18, 20]. Data from different populations like Iran [52], Turkish [38], and Germany [53] also stated that lower first and second premolars vary more concerning roots and canals in men than women. But these results were in contradict the study of Sert and Bayirli [54] which says that more variations are reported in females than males, it is most likely due to the destruction of the root canal caused by staining technique and decalcification. However, in our study, no statistically significant difference was reported between mandibular premolars and gender. Similar findings were reported among the Turkish population [52]. Even though the root canal morphology varied across the genders, these changes were not statistically significant. According to our findings, males were more likely than females to have two roots and canals in mandibular first and second premolars. The method of canal morphology assessment, the target ethnic groups, and other factors could all have an impact on the variations in the results.

As age increases and secondary dentine continues to deposit, it can alter the structure of preformed root canals. Incomplete root formation in a population below 15 years of age can also show altered root canal morphology [18]. In the present study, the 31–40 years population presented a higher incidence of ^1^ TN ^1^ followed by 21–30 years in the left mandibular first premolar. Whereas the distribution of left mandibular 2nd premolar with ^1^ TN ^1^ classification was higher among 21–30 years than 31–40 years. Right mandibular 1st and 2nd premolar with ^1^ TN ^1^ classification was higher among 31–40 years, followed by 21–30 years of the studied population. When comparing the characteristics of mandibular premolars with age, a statistically significant difference is reported. Similar findings were reported in Belgian and Chilean populations [21]. Overall, the mandibular first and second premolars (both sides) have different root canal morphologies, and the number of canals on one side does not necessarily determine the shape of the canals on the opposing side.

## Limitations

There are a few limitations that need to be addressed. Due to the fact that this research was conducted at a single location, the size of the sample should have been greater. Further, this study is a retrospective analysis with variable voxel scans and field sizes, which may have an impact on the results. A more precise estimation of the prevalence of this distribution in the Saudi population could be obtained by multicentre research with bigger sample sizes. In addition, the CBCT employed in this research has a poorer spatial resolution than micro- and nano-CT, which may have played a role in the findings.

## Conclusion

The higher number of mandibular premolars had type I (^1^ TN 1) canal configuration, which was higher among males. According to our findings, the difference in canal numbers between the left and right mandibular premolars was statistically significant concerning age but not concerning gender. Given that gender and age were disregarded as potential contributing factors. The CBCT imaging provides thorough details regarding the root canal morphology of mandibular premolars. These findings could support diagnosis, decision-making, and root canal treatment, for dental professionals. Future research with a bigger sample size and other reformatting methods in micro-computed tomographic imaging will be beneficial to comprehend the frequency of lateral canals and intracanal anastomoses in mandibular premolars.

## Data Availability

The datasets used and/or analysed during the current study are available from the corresponding author on reasonable request.

## Abbreviations

CBCT: Cone-beam computed tomography.
MicroCT: Micro-computed tomography
